# Exploring Protein Cavities through Rigidity Analysis

**DOI:** 10.3390/molecules23020351

**Published:** 2018-02-07

**Authors:** Stephanie Mason, Brian Y. Chen, Filip Jagodzinski

**Affiliations:** 1 Department of Computer Science, Western Washington University, 516 High Street, Bellingham, WA 98225, USA; stephanie.mason@wwu.edu; 2 Department of Computer Science and Engineering, Lehigh University, 19 Memorial Drive West, Bethlehem, PA 18015, USA; Chen@cse.lehigh.edu

**Keywords:** protein, cavity, rigidity analysis

## Abstract

The geometry of cavities in the surfaces of proteins facilitates a variety of biochemical functions. To better understand the biochemical nature of protein cavities, the shape, size, chemical properties, and evolutionary nature of functional and nonfunctional surface cavities have been exhaustively surveyed in protein structures. The rigidity of surface cavities, however, is not immediately available as a characteristic of structure data, and is thus more difficult to examine. Using rigidity analysis for assessing and analyzing molecular rigidity, this paper performs the first survey of the relationships between cavity properties, such as size and residue content, and how they correspond to cavity rigidity. Our survey measured a variety of rigidity metrics on 120,323 cavities from 12,785 sequentially non-redundant protein chains. We used VASP-E, a volume-based algorithm for analyzing cavity geometry. Our results suggest that rigidity properties of protein cavities are dependent on cavity surface area.

## 1. Introduction

Many biological functions performed by proteins depend on the size, shape, and chemical properties of cavities on the molecular surface. For example, the largest and deepest cavity in a protein is frequently a ligand binding site and the shape of a cavity is a target for ligand design [1]. The size and geometry of cavities have proven useful in making predictions about protein–protein interactions, protein druggability [2,3], and binding specificity [4]. The discovery of these trends stem from many comprehensive surveys that have correlated cavity structure, chemistry, and evolution [5] to biochemical function. While many characteristics of cavities have been carefully surveyed, cavity flexibility is not a property that is provided in protein structure data, and thus it can be more difficult to survey. Nonetheless, flexibility is well-known to play a key role in function. Binding cavities, such the protein kinase binding site [6], can occupy active and inactive conformations. Thus, changes in conformation are critical to understanding function. Some cavities may retain their shape in the unbound state, as many STARt domains do, whereas other may collapse [7]. Cavity flexibility and its impact on function is anecdotally established in many cases, but a systematic effort to survey it in protein cavities has not been performed.

To fill this gap, this paper surveys the flexibility of cavities from a large selection of protein structures using a property called rigidity. We say that an atom is part of a rigid cluster of atoms if it has no degrees of freedom due to the geometry of bonds and contacts. Atoms between rigid clusters are flexible. Identifying rigid components of a molecular model of a protein can thus comprehensively characterize the flexibility of a molecule in some regions and the lack of flexibility elsewhere. We use an efficient combinatorial algorithm [8,9,10] to identify rigid clusters in both functional and nonfunctional cavities. To identify cavities, we use VASP-E, a volumetric algorithm for finding and comparing cavities. Together, the outputs of these programs were analyzed to understand the relationships between rigidity and cavity size, residue content, and evolutionary significance [11,12,13]. Our survey is the first effort to evaluate the relationship between rigidity and fundamental cavity properties.

### Related Work

Previous research has found that the active site frequently resides in the protein cleft with the largest volume [3]. Additionally, when the largest surface cavity in a protein is the active site, that cavity tends to be significantly larger than other clefts on that protein. However, when the second largest cleft was the active site, the two largest clefts tended to be similar in size. This trend established cavity size as an important predictor of protein function. Other studies have extended that work, further establishing protein cavities as an area of particular interest.

Experiments elucidating the roles of cavities have also been conducted in vitro on a variety of physical proteins. For example, Musah et al. studied the binding thermodynamics of the cytochrome complex [14], and report that that cavities exhibit strong specificity for heterocyclic cations. Among 18 X-ray resolved structures with bound molecules, Musah showed that cavities induced by the mutations were able to exclusively bind specific molecules. For these reasons, it is clear that biophysical characteristics other than shape are crucial for cavity function.

Such characteristics can be altered by mutation. Bade et al. studied cytotoxic T lymphocytes and peptide-human leukocyte antigen complexes [15]. They explored the role of single amino acid substitutions, and identified six pockets which play a specificity role in restricting antigen binding, and that polymorphism affects which cavities the antigen is compatible with.

Binding properties can also be affected by secondary structure. In work by Chan et al., synthetic C peptides were engineered to study drug targets of the HIV-1 gp4 envelope protein [16]. Crystal structures of the core of gp41 were resolved, which showed that 3 C helices pack against a central coil when the protein is in its function-activation conformation, and that the helices make strong contacts with hydrophobic cavities on the surface of the coiled coil.

One important influence on cavity function is conformational flexibility. Binda et al. studied monoamine oxidase B (MAO B), which due to its role in a variety in neurological disorders, is a common target for antidepressants and neuroprotective drugs [17]. Several secondary structures of the oxidase were identified to play key roles in the protein’s binding affinity with substrates. The active site was identified as a 420Å^2^ hydrophobic cavity interconnected with a second cavity of almost the same size. Binda’s work has resulted in a greater understanding of the catalytic mechanism of the oxidase, and subsequent drug design studies often consider the induced fit of ligand–enzyme interactions [18].

Studies at the University of Cambridge have elucidated the importance of flexibility in cavity function for an entire class of proteins. Elliott et al. studied the serpin family of serine proteinase inhibitors to improve understanding of their roles in the inflammatory and fibrinolytic cascades [19]. Five cavities were identified as potential drug targets, which were hypothesized to be associated with conformational changes of the proteinase inhibitor family of molecules. Knowledge of the cavities involved has enabled the rational design of drug agents to prevent conformational transitions and pharmacologically control the debilitating diseases they cause. It is clear that flexible mechanisms in protein cavities can play an important role in function, so surveying cavity flexibility at a large scale may provide informative insights into functional mechanisms in general.

The classic approach to assessing flexibility relies on the simulation of protein motion using energy functions [20,21,22]. Such approaches form the bedrock of many biophysical studies, making them totally reliable, but they are computationally time consuming and they offer more detail than is required for a survey. In particular, energy-based methods can provide the directions in which each atom has degrees of freedom, but for this survey, simply knowing whether an atom has no degrees of freedom is sufficient. The methods used here provide this information in seconds, enabling us to generate survey-scale flexibility data.

Many algorithms have been developed to find protein cavities for such a survey. Such tools often leverage insights from machine learning. For example, SCREEN utilizes a Random Forest approach to identify druggable cavities. SCREEN analyzes surface cavities of nonredundant proteins crystallized with drugs [23]. MetaPocket 2.0 is a popular web service that predicts drug binding sites with approximately 75% accuracy [24]. Fpocket detects and identifies ligand binding pockets through the use of alpha spheres and Voronoi tessellation [25].

## 2. Results

**Figure 1:**
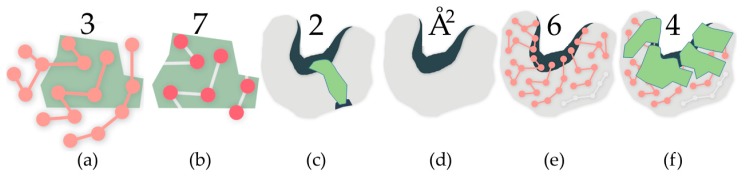
In all subfigures, the green polygons represent rigid clusters of atoms. **Rigid cluster-based metrics**: (**a**) Residues in a rigid cluster; (**b**) Atoms in a rigid cluster; (**c**) This rigid cluster has participation in two cavities, a large one and a small one; **Cavity-based metrics**: (**d**) The surface area of the cavity; (**e**) The count of residues with participation in the cavity, determined by shared atoms; (**f**) The count of rigid clusters with participation in the cavity, determined by shared atoms.

Our results are organized into two sections. First, we examined rigidity as it occurs in whole cavities, plotting the number of rigid clusters in a cavity to cavity surface area and evolutionary significance. Cavity surface area, measured in square angstroms, is assessed by generating a molecular surface and computing the total surface area in the cavity. Evolutionary significance, which we measure using data from the Evolutionary Trace Server, quantifies the importance of an amino acid to the function of a protein. Second, we examined rigid clusters of individual atoms, relating rigid clusters to the number of atoms in the cavity, cavity participation, and evolutionary significance. Cavity participation is the number of cavities that a rigid cluster is a part of. See Figure 1 for an overview of the types of rigidity- and cavity-based metrics that we surveyed.

### 2.1. Protein Selection

Our dataset was constructed of 12,785 non-redundant protein chains with less than 30% pairwise sequence identity. We generated this collection using the PDB web service (see Section 4.1.4). These chains were found to contain 593,295 cavities of any size and 867,530 rigid clusters. Given that datasets of this size are difficult to visualize in a single plot, unless explicitly noted, the plots in this manuscript show only 1000 randomly selected members of the dataset to increase visual clarity while remaining consistent with trends found in the larger dataset.

### 2.2. Properties of Cavities

**Figure 2:**
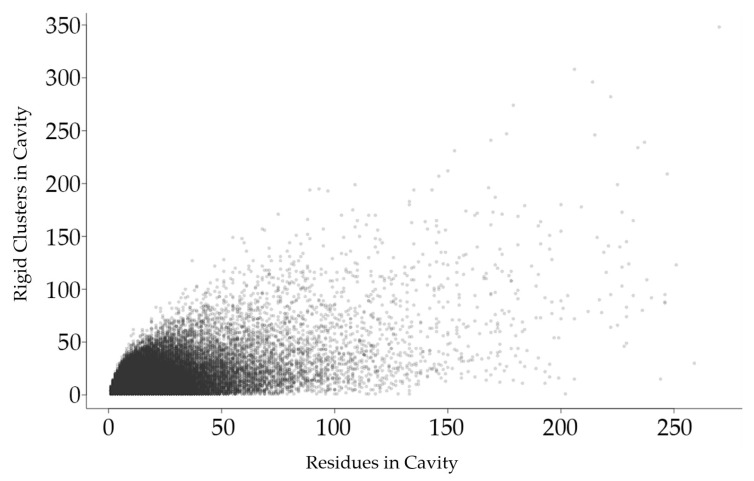
Each point in this plot represents a protein cavity, relating the number of rigid clusters in a cavity (vertical axis) relative to the number of amino acids in the cavity (horizontal axis). All cavities in all 12,785 chains in the dataset are shown.

**Figure 3:**
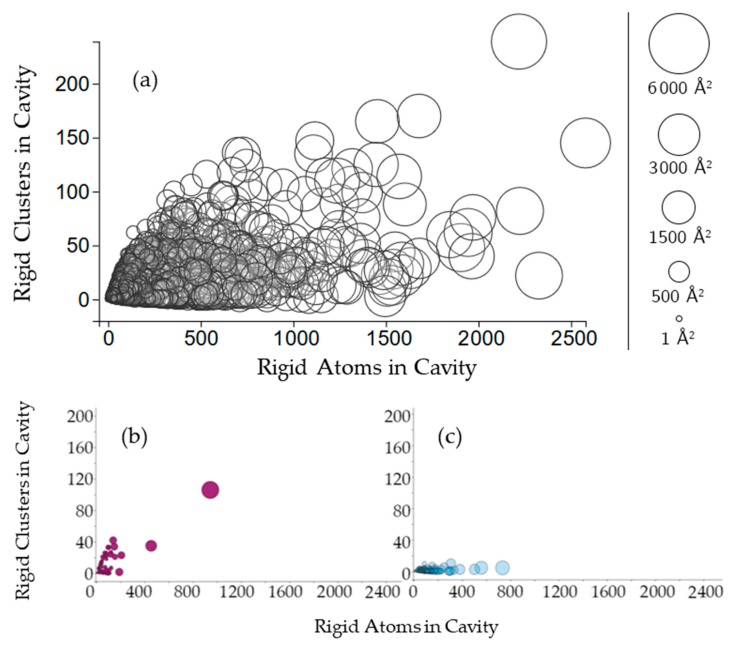
(**a**) The plot indicates the relationship between the number of rigid clusters in a cavity versus the number of rigid atoms in a cavity. Cavities are shown as circles, where the center of the circle plots the number of rigid clusters in the cavity and the number of rigid atoms in the cavity. The radius of the circle indicates the cavity surface area (Å^2^); (**b**,**c**) Cavities of two hydrolases, BipA and alpha-transglucosylase (PDB 3e3x, chain A, PDB 4ba0, chain A), on the same axes.

**Figure 4:**
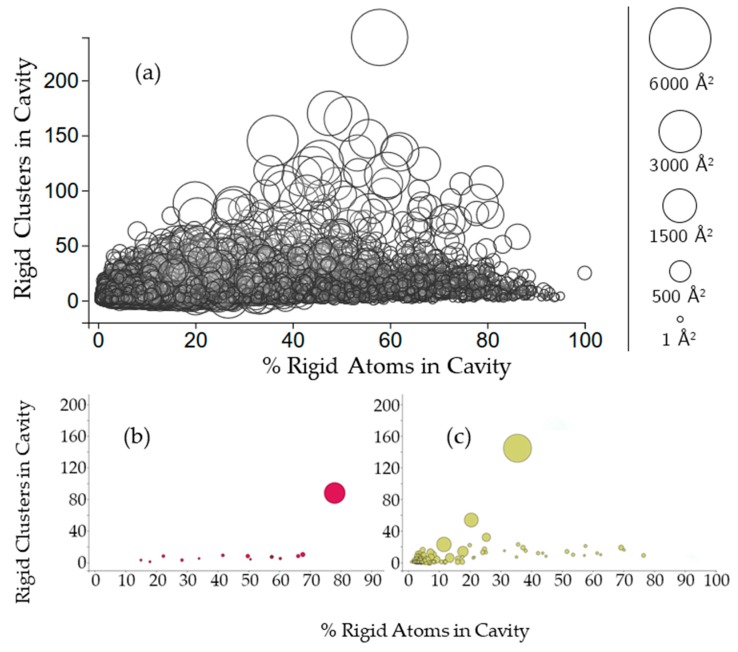
(**a**) The relationship between the size of cavities, the number of rigid clusters in them, and the percentage of rigid atoms. Cavities are shown as circles, where the center of the circle plots the number of rigid clusters in the cavity versus the percent of rigid atoms from those clusters in the cavity. The radius of the circle indicates the cavity surface area; (**b**,**c**) Cavities of a membrane protein and a plasminogen (PDB 2k3m, chain A, PDB 4dur, chain A).

**Figure 5:**
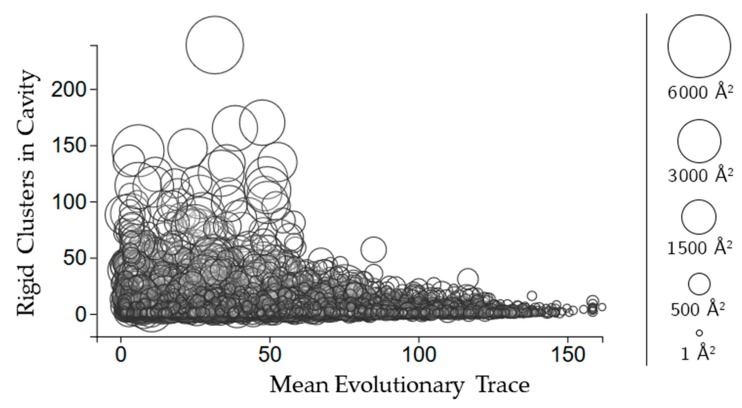
The relationship between evolutionary trace and the number of rigid clusters in a cavity. Circles represent a cavity, with the size being proportional to the surface area. The center of each point corresponds to the number of rigid clusters in the cavity (vertical axis) and the mean evolutionary trace score calculated for all of the residues in the cavity (horizontal axis).

We first survey the relationship between cavity rigidity and other cavity properties. Figure 2, Figure 3, Figure 4 and Figure 5 plot the relationship between the number of rigid clusters in a dataset of cavities with the number of residues in the cavity, the number of rigid atoms, and the evolutionary significance of amino acids in the cavity.

#### 2.2.1. Relationship between Rigid Clusters and Residues

Figure 2 shows that the total number of rigid clusters in a cavity has a weakly positive correlation with the number of residues in a cavity, with a correlation coefficient of 0.576.

#### 2.2.2. Protein Cavities: Rigid Clusters and Rigid Atoms

Cavities with more rigid clusters tend to have more rigid atoms overall (Figure 3a), with a correlation coefficient of 0.498. The nonlinearity of this relationship suggests variability in the rigidity of cavities. In a single protein chain, cavities with greater surface area will tend to have more rigid clusters and more rigid atoms. This relationship is consistent for almost all of the chains plotted. Two examples can be seen in chain A of structures 3e3x and chain A in 4ba0 (Figure 3b,c).

To further investigate this rigid cluster participation, we calculated the percent of rigid atoms in cavities versus the percent of rigid atoms that are not part of a cavity for all of the rigid clusters with participation in the cavity. Figure 4 shows that the number of rigid clusters in a cavity is not correlated with the percent of the rigid atoms in the cavity. The percent of a protein’s rigid atoms that are in a cavity has a distribution approaching normal (with plenty of exceptions, particularly in individual chains such as 2k3mA). Running a nonlinear least squares fit analysis on the data yielded a correlation coefficient of 0.462. Larger cavities are clustered more closely to 50% rigid atom participation than they are towards the extremes. A few individual chains (2k3mA and 4durA) show a typical trend of the largest cavity to have the most rigid clusters. However, there are exceptions. For 2k3mA, the largest cavity appears to have overall more rigid involvement (with about 80% of the rigid atoms being contained in the cavity), whereas for 4durA, the largest cavity only has about 35% of the rigid atoms.

#### 2.2.3. Protein Cavities: Evolutionary Significance

Evolutionary trace is a score of how conserved sequences are in a protein [11]. We use the mean real value score (see methods for details) of all of the scores for the residues in each cavity. Figure 5 shows that larger cavities have lower mean evolutionary trace scores overall. There is no clear relationship between how many rigid clusters there are in a cavity and the mean evolutionary trace.

### 2.3. Properties of Rigid Clusters in Cavities

We also surveyed the relationship between individual rigid clusters and other cavity properties. These relationships include the relationship between rigid clusters and cavity size, cavity participation, and evolutionary significance.

#### 2.3.1. Rigidity Properties of Cavities: Cavity Size

**Figure 6:**
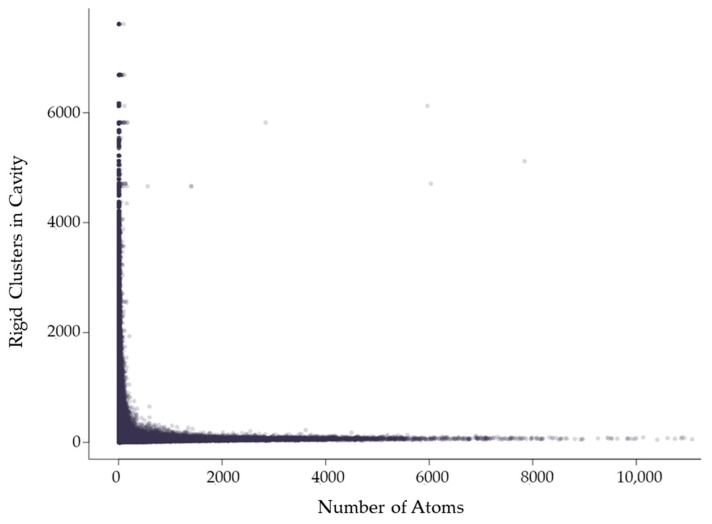
The mean cavity size in which a rigid cluster has participation relative to the number of atoms in the rigid cluster, which corresponds to its size. Each point indicates a rigid cluster. This plot contains all 12,785 chains in the dataset.

**Figure 7:**
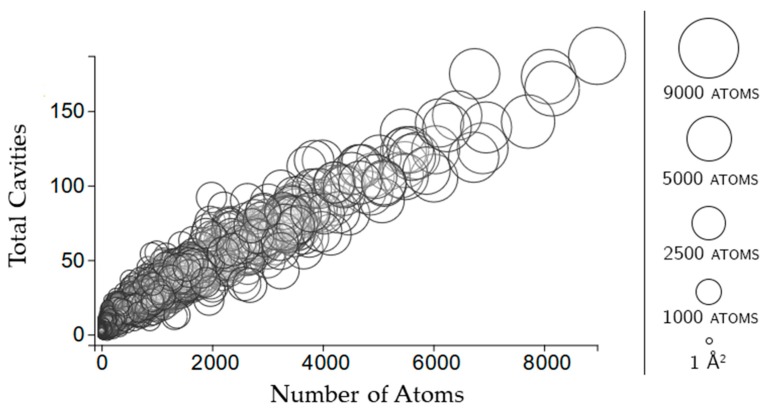
Shown here is the same subset of 1000 chains as the cavity plots. Circles indicate a rigid cluster. The size of the circle and its *x*-coordinate indicate the size of the rigid cluster based on the number of atoms contained in it. The *y*-coordinate indicates the number of cavities with which that rigid cluster shares atoms.

Rigid clusters are composed of a set of atoms. Because of this, a rigid cluster with a larger number of atoms indicates a larger rigid cluster, and a small number of atoms indicates a small rigid cluster. Figure 6 shows that as rigid clusters grow larger in size, the mean cavity size that the cluster is a member of quickly decreases. This suggests that even if a large rigid cluster has participation in a large cavity, it also necessarily has participation in many smaller cavities. Only small rigid clusters can have a large mean cavity size, presumably because they are small enough that they cannot physically span enough of a protein to have participation in multiple cavities. This is confirmed by Figure 7, which shows that larger rigid clusters, indeed, predictably overlap with more cavities. The total number of cavities a rigid cluster participates in correlates strongly with its size, having a correlation coefficient of 0.943.

#### 2.3.2. Rigidity Properties of Cavities: Cavity Participation

**Figure 8:**
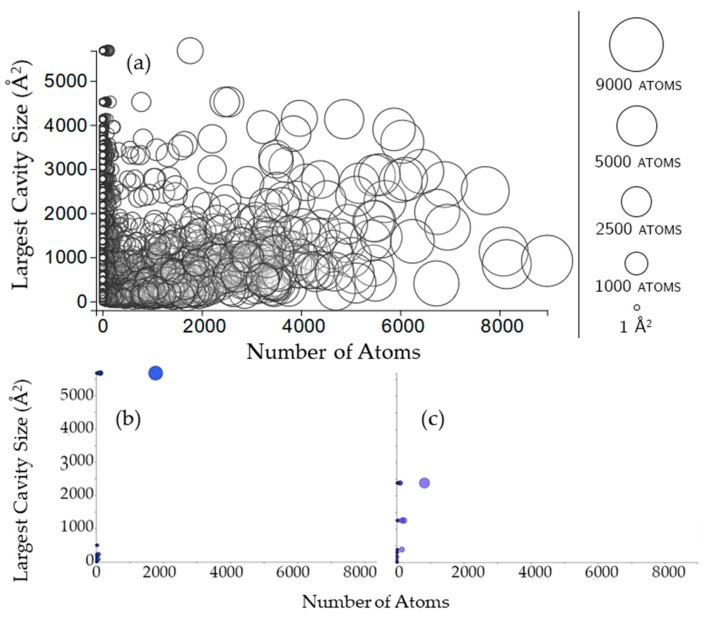
(**a**) Circles here are rigid clusters, with the size of the circle and its *x*-coordinate both corresponding to the number of atoms in the rigid cluster. The *y*-coordinate corresponds to the surface area of the largest cavity for which that rigid cluster has participation, in Å^2^; (**b**,**c**) Rigid clusters in the cavities of a Small Ubiquitin-like Modifier (SUMO) protein and inositol polyphosphate (PDB 3kyd, chain B, PDB 1inp, chain A).

The size of a rigid cluster does not seem to have any influence on the size of cavities that the rigid cluster participates in (Figure 8). For some chains (such as 3kyd, Figure 8b), all of the rigid clusters appear to be in the largest cavity. For other chains (such as 1inp, Figure 8c), this is not the case.

#### 2.3.3. Rigidity Properties of Cavities: Evolutionary Trace Scores

**Figure 9:**
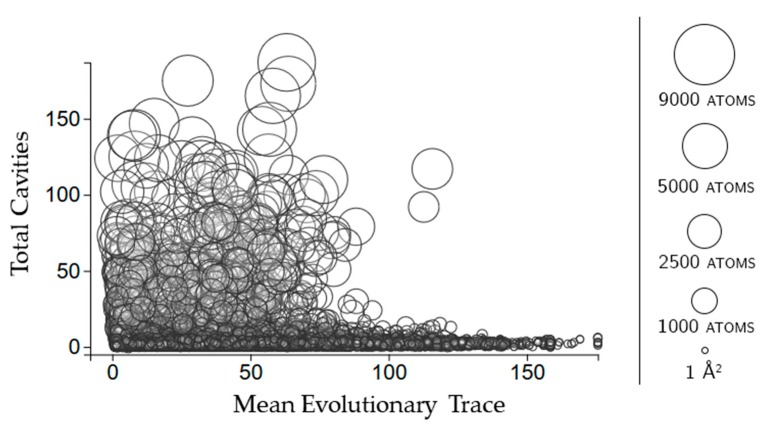
The relationship between the number of cavities in which a rigid cluster has participation and the mean evolutionary trace of all the residues in that rigid cluster. Circles represent rigid clusters, with the size being proportional to the number of atoms in the cluster.

Similar to how we found the mean evolutionary trace of cavities, we found the mean evolutionary trace of rigid clusters by averaging the rvet score across all of the residues contained in a rigid cluster. Small rigid clusters run the entire range of evolutionary trace scores, from being highly conserved to not very conserved (Figure 9). Large rigid clusters are almost exclusively restricted to lower evolutionary mean evolutionary trace scores. This suggests that there is a limit to how evolutionarily conserved the sequences comprising rigid clusters can be, with low conservation not being unusual. The total number of cavities that a rigid cluster is participating in appears to have no effect on its evolutionary trace score.

## 3. Discussion and Conclusions

For this work, we surveyed the flexibility and biomolecular properties of 120,323 surface cavities among 12,785 sequentially non-redundant protein chains. We determined flexibility by identifying rigid clusters of atoms, calculated using an efficient combinatorial algorithm that outputs sets of atoms among which there are no trivial degrees of freedom. The biomolecular properties and metrics that we explored include cavity surface area, which we measured using VASP-E, the count and type of atoms and residues in each cavity, as well as the evolutionary significance of residues as inferred using data from the Evolutionary Trace Server. We generated plots for all pairwise combinations of these metrics for our dataset, and report in this paper on those plots for which the metrics showed a significant correlation, or for which there was a noteworthy lack of correlation.

Among several relationships involving metrics for which there was a lack of a correlation, we observed that cavity size and the count of rigid atoms that are on the immediate cavity surface varies significantly. Some of the largest cavities are comprised of a surface made up of 80% of the atoms that are members of the rigid clusters in the cavity. Other protein chains have their largest cavities with a cavity surface that is composed of rigid atoms that make up only 35% of all the rigid atoms of the rigid cluster that have membership in the cavity. Biologically speaking, this shows that the extent into a protein’s core away from the surface of the cavity that a rigid cluster extends varies. Additionally, we observed that it is not unusual for a rigid cluster to be made up of residues that are not evolutionarily conserved. Lastly, a correlation that we observed is that even if a large rigid cluster has participation in a large cavity, it also necessarily has participation in many smaller cavities.

Although for this work we did not conduct wet-lab validation studies to elucidate the implications on biological processes that these correlations might have, there are takeaways nonetheless. One is that certain rigidity properties of cavities, such as the distance away from a cavity’s surface and towards the protein’s core that a rigid cluster extends, are not universal among all protein chains. Another is that residues in a rigid cluster that are part of a cavity are often not conserved. Such information might inform protein engineering studies on how to engineer a pharmaceutical drug with increased efficacy due to its leveraging a certain rigidity property of a cavity.

## 4. Materials and Methods

### 4.1. Computational Pipeline

**Figure 10:**
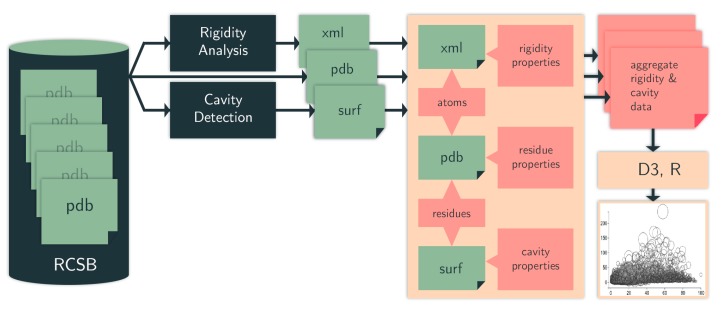
Computation pipeline. We identify cavities (surf data) among protein chains in PDB files (pdb data). We use an efficient rigidity analysis approach to identify rigid clusters of atoms in a protein (cluster data). The structure, rigidity, and cavity files are aggregated to generate details of the rigidity properties of the cavities. From the aggregate metrics, we plot various cavity-rigidity-atom properties. RCSB = Research Collaboratory for Structural Bioinformatics Protein Data Bank (PDB).

Our computation pipeline (Figure 10) is made up of custom-built scripts that integrate rigidity analysis software and cavity detection tools. Some of these tools we have designed ourselves, while others are off-the-shelf.

#### 4.1.1. Identifying Cavities

The software *VASP-E* [26] was used to identify cavities and to calculate their surface areas. The surface area is the sum of the areas of all triangles defined to be on the surface based on the molecular surface area [4]. Molecular surfaces are defined as C1-smooth surfaces that surround the atoms of a protein. A molecule’s surface is generated conceptually by rolling a ball along the surface, and defined by the surface of the region of space that ball cannot occupy [27]. The residues that participate in each cavity were also identified as the set of amino acids closest to the cavity surface. Specifically, the surface is defined as a mesh of triangles and every triangle has an atom of the structure closest to it. That atom is part of some amino acid, which is added to a list.

#### 4.1.2. Rigidity Analysis

**Figure 11:**
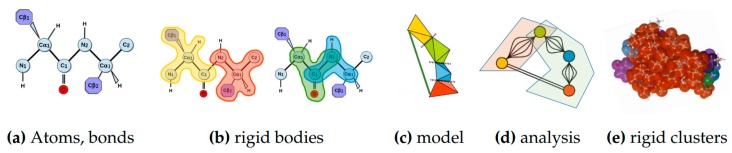
Rigidity Analysis. Atoms from a PDB file and bonds among them (**a**) determine rigid bodies (**b**), the smallest rigidity components in a molecule. A mechanical model (**c**) representing the degrees of freedom among the rigid bodies is converted to an associated graph (**d**) on which rigidity analysis is performed. Its output is used to identify rigid clusters of atoms in the protein (**e**).

Rigidity analysis [8,28] is a fast graph-based method that provides information about the flexibility of proteins, which are known to contain regions of varying degrees of rigidity [29]. In rigidity analysis, atoms and their chemical interactions are used to construct a mechanical model of a molecule. The mechanical model represents a structure that permits hinge-like motions arising from rotations of some peptide dihedral angles (phi and psi), but not others (specifically omega), in the atoms of a molecule. A graph is constructed from the mechanical model in which each body is associated to a node, a hinge between two bodies to five edges, and a bar is associated to an edge. The number of edges between any two nodes in the associated graph represents the degrees of freedom between the atoms that make up the nodes’ bodies in the mechanical model. Pebble game algorithms [9] are used to analyze the rigidity of the associated graph. The results of a rigidity analysis, which identifies rigid nodes in the graph among which there are no degrees of freedom, can be used to infer the rigid and flexible regions of the mechanical model, and ultimately the protein, that the associated graph represents. See Figure 11 for an overview of rigidity analysis.

**Figure 12:**
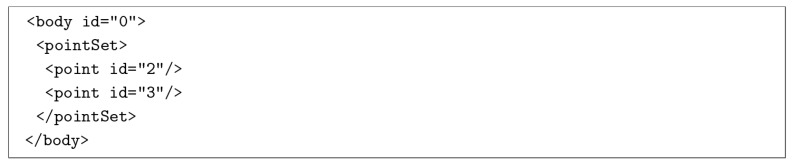
Rigidity Analysis output file in XML format. Point id values correspond to atom IDs in a PDB file. All of the points in a body represent the atoms having communal membership in a rigid cluster. Each body has an id, to distinguish it from the other rigid clusters.

In this work, we use the freely available KINARI rigidity software [10] to calculate the rigidity properties of each protein chain in our data set. The rigidity analysis output is an xml file containing information about the identified rigid clusters of atoms (Figure 12). The following is a sample output of the rigidity analysis of a protein, which identifies atoms 2 and 3 (the IDs from the PDB file) as being members of the rigid body with ID 0.

#### 4.1.3. Evolutionary Trace

In addition to the analysis run in Figure 9, we downloaded the evolutionary trace files for all of the chains in our study from the Evolutionary Trace Server available from the Lichtarge Lab [11]. Evolutionary trace is a method of estimating the evolutionary importance of individual protein residues. A real value score is assigned to each residue by comparing variations in sequences at that position and correlating them with an evolutionary tree. Higher scores will correspond to the root of the tree with little variation, while lower scores will correspond to leaves of a tree with high variation [6]. We averaged the real value score for all of the residues in a cavity or rigid cluster and output both the mean and median scores into the aggregate JSON/TSV files.

#### 4.1.4. Data Selection, Aggregation, and Analysis

For our analyses, we used the non-redundant set of PDB IDs provided by the PDB. Several lists are provided that remove similar sequences from the complete set of PDB structure files using BLASTClust at different levels of sequence similarity. We used the list containing 30% BLAST identity, which contains 28,326 IDs. PDB files contain structural information about proteins, including atom IDs and residue IDs. Rigidity analysis provides outputs containing atom IDs of rigid clusters. Cavity detection creates an output containing a list of residue IDs associated with cavities. Evolutionary trace files contain scores for residues IDs. We created a script to use these atom and residue IDs to associate cavities to rigid clusters based on overlapping atoms and to calculate a large number of metrics to explore.

Because we relied on several different programs or software tools, at each step of our pipeline there were PDBs for which an analysis could not be completed. Some PDBs had simple formatting or naming errors that resulted in an incomplete rigidity analysis or cavity detection. For some others, evolutionary trace data was not available. To aggregate the metrics of interest, we needed complete outputs from each of these individual programs. This resulted in a smaller dataset than the original RCSB 30% list, but one of substantial-enough size for meaningful data analysis.

We had final, aggregated data for 12,785 chains, which we used to generate static plots in R. To explore the data as thoroughly as possible, we created interactive scatter plots in D3 with an additional size dimension (the size of the points, shown as spheres, in our plots). These plots became very noisy to the point of being uninterpretable, so we further reduced the data set for these plots down to 1000 unique chains. Comparing these plots to those containing the full set showed that there was not an appreciable difference. Using D3 allowed us to investigate the relationships between many different unique metrics, many of which did not yield any useful information. We pared down those plots to those included in this manuscript, and focused on the metrics that either showed some significant correlation, or were otherwise noteworthy in their lack of correlation.
